# Multiplex real-time RT-PCR method for the diagnosis of SARS-CoV-2 by targeting viral *N*, *RdRP* and human *RP* genes

**DOI:** 10.1038/s41598-022-06977-z

**Published:** 2022-02-18

**Authors:** Huseyin Tombuloglu, Hussein Sabit, Hamoud Al-Khallaf, Juma H. Kabanja, Moneerah Alsaeed, Najat Al-Saleh, Ebtesam Al-Suhaimi

**Affiliations:** 1 Department of Genetics Research, Institute for Research and Medical Consultations (IRMC), Imam Abdulrahman Bin Faisal University, P.O. Box 1982, Dammam, 31441 Saudi Arabia; 2 Department of Pathology and Laboratory Medicine, King Fahad Specialist Hospital, Dammam, Saudi Arabia; 3 Department of Family and Community Medicine, King Fahad Hospital of the University, Imam Abdulrahman Bin Faisal University, Dammam, 31441 Saudi Arabia; 4 Department of Biology, College of Science and Institute of Research and Medical Consultations (IRMC), Imam Abdulrahman Bin Faisal University, Dammam, 31441 Saudi Arabia

**Keywords:** Biological techniques, Molecular medicine

## Abstract

Corona Virus Disease 2019 (COVID-19) is a disease caused by severe acute respiratory syndrome coronavirus 2 (SARS-CoV-2). This pandemic has brought the world to a standstill and threatened human lives. Many methods are known to date to detect this virus. Due to their relative sensitivity, polymerase chain reaction (PCR)-based assays are the most frequently applied and considered the gold standard. However, due to the rapid mutation rate of the viral genome and the emergence of new variants, existing protocols need to be updated and improved. Designing a fast and accurate PCR-based assay is of great importance for the early detection of this virus and more efficient control of the spread of this disease. This study describes a fast, reliable, easy-to-use, and high-throughput multiplex SARS-CoV-2 RT-PCR detection method. The assay was designed to detect two viral genes (*N* and *RdRP*) and a human gene (*RP*) simultaneously. The performance and the sensitivity of the assay were tested in 28 SARS-CoV-2 positive samples and compared with commercial kits, which showed 100% positive percent agreement with a limit of detection (LOD) value of 1.40 and 0.81 copies/µL or 35.13 and 20.31 copies/reaction for *RdRP* and *N* genes, respectively. The current assay is found accurate, reliable, simple, sensitive, and specific. It can be used as an optimized SARS-CoV-2 diagnostic assay in hospitals, medical centers, and diagnostic laboratories as well as for research purposes.

## Introduction

Severe acute respiratory syndrome coronavirus 2 (SARS-CoV-2) belongs to the subfamily *Orthocoronavirinae* of the family *Coronaviridae* of the order *Nidovirales*. As of December 18, 2021, about 273 million cases and almost 5.35 million deaths are related to SARS-CoV-2^1^. The pandemic that started in late 2019 and continued to increase in 2020 has forced scientists and clinicians to develop new methods for diagnosis and treatment^2^. In addition to some known methods, studies to develop faster, easier, and more reliable methods for SARS-CoV-2 diagnosis continue intensively. These methods are mainly divided into nucleic acid-based amplification tests and serological tests. The nucleic acid-based amplification methods, such as rRT-PCR, are based on the amplification of viral RNA^3^. On the other hand, serological tests are based on detecting either the proteins of the COVID-19 virus or human antibodies generated in response to infection such as the immunoglobulin type M (IgM) or immunoglobulin type G (IgG). The most important disadvantage of serological tests is their limited sensitivity to early detection^4^. In general, the development of specific antibodies against the virus begins after the first week, and IgM and IgG production occurs mostly in the second week^5^. Therefore, the sensitivity of serological tests is limited in the acute stage of infection. Another drawback is the possibility of similar antibody responses to the viruses in the same or close families and the possibility of cross-reactivity. This possibility is a serious concern as most human coronaviruses are antigenically close related to each other. Therefore, the WHO and CDC do not recommend the use of point-of-care immunodiagnostic tests for clinical decision-making. Instead, serological tests can be used for research or clinical support purposes^6–8^.

On the other hand, the reverse transcription polymerase chain reaction (RT-PCR) is the most common and accepted as the gold standard method for the viral detection^9^. Although RT-PCR is a standard assay, it might produce false-negative and false-positive results due to lack of specificity and sensitivity^9–11^. According to Fang et al.^12^, RT-PCR was able to detect only 71% (36/51) of SARS-CoV-2 infections. This may be due to the low sensitivity of the test, low patient viral load, or inappropriate clinical sampling^12^. The probability of obtaining a true positive result decreases with time from symptom onset. Nasopharyngeal and oropharyngeal RT-PCR tests performed on the 10th day of the first symptom have a 25% and 47% chance of being false-negative, respectively^13^. Reports showed that patients with negative initial tests became positive after 3–5 repeated swabs^14^. The rate of false-negative result changes in between 1 and 30%^15^. Therefore, the sensitivity of this method should be improved. For this purpose, multiplex PCR protocol is proposed that targets multiple genes in the same reaction. By doing so, at least two viral genes are targeted that help to increase the probability to catch the virus especially in the patients having low viral load. In addition, a human housekeeping gene can be added as an internal control to prevent false-negative results that are due to inefficient sampling.

The genome of SARS-CoV-2 contains 14 ORFs encoding for 27 proteins^16^. The genome of this virus is composed of four major structural genes that are translated to spike protein (S), small envelope protein (E), matrix protein (M), and nucleocapsid protein (N)^16^, along with some accessory genes. The surface glycoproteins (S1 and S2) are responsible for binding to the ACE2 (Angiotensin-converting enzyme 2) receptors on the host cell allowing the virus to invade, where S1 bind to the ACE2 receptor and S2 fuses with the host cell membrane^17,18^. In addition, the genome harbors genes encoding non-structural proteins (Nsp) such as RdRP (Nsp12), the RNA-dependent RNA polymerase enzyme responsible for the replication of the viral genome^19,20^.

RT-PCR tests have been developed to target *RdRP*, *E*, *N*, or *S* genes^10,21,22^. Among these, those targeting *RdRP* gene were found to be the ones with the highest analytical sensitivity^9,23^. In addition, the human ribonuclease P (*RNase P* or *RP*) gene (responsible for the processing of tRNA molecules) is used as an internal control in multiplex RT-PCR protocols recommended by WHO and CDC^24–26^. There is also a need for studies to compare between recently developed assays based on their reproducibility, and sensitivity. Therefore, in addition to the experimental design, determining which genes to be targeted by the assay, are also extremely important goals to achieve when designing molecular testing protocols. Although multiplex RT-PCR method helps to improve the specificity, the design of multiplex primers and probe sets is very critical for the PCR efficiency. The formation of self-dimer or hetero-dimer structure reduce the target specificity and may lead to misinterpretation of the results. Therefore, the experimental design as well as the selection of the best primer and probe sets are crucial and needs standardization. Together with, real-time RT-PCR results may be affected by variations in viral RNA sequences^27^. A recent study estimated the nucleotide mutation rate of the SARS-CoV-2 genome as 6.677 × 10^−4^ substitution per site per year^28^, which lead loss of the assay sensitivity as shown by Peñarrubia et al.^29^. The emergence of new variants and the high mutation rate of the viral genome require updating existing diagnostic tests.

In this study, a multiplex real time RT-PCR (rRT-PCR) assay was designed and evaluated for the diagnosis of SARS-CoV-2 including the most recent variant of concerns (VOC). The study exhibits primer sets specific to SARS-CoV-2 and the accompanying optimized reaction conditions. The developed assay simultaneously detects viral *N*, *RdRP* and human *RP* genes in the same rRT-PCR reaction. The clinical performance of the test was screened with RNA samples from SARS-CoV-2 positive patients.

## Materials and methods

### SARS-CoV-2 genome sequences

In total, 344 SARS-CoV-2 genomes belongs to variants of concern (VOC) including alpha (B.1.1.7), B.1.351 (Beta), P.1 (Gamma), and B.1.617.2 (Delta) were analyzed. The sequences were retrieved from GISAID database^30^ and chosen by covering all continents including Europa, North and South America, Asia, Africa, and Oceania. The sequences were aligned by using MAFFT software (https://mafft.cbrc.jp/alignment/server/) with default settings^31^. Then, the interested genome regions harboring the primer target sites for *N* and *RdRP* genes were selected and a possible substitution or single nucleotide polymorphism at the primer binding sites were screened by using JalView (v2.11.1.3) program^32^ (Supplementary Figs. S4 and S5). The consensus sequences (100% alignments) corresponding to the target genes were selected for primer design. The primers were synthesized by Molequle-On (Auckland, New Zealand) and purified by high-performance liquid chromatography.

### Multiplex primer/probe design

To design the most suitable multiplex primers, PrimerPooler^33^, PrimerPlex (http://www.premierbiosoft.com/primerplex/index.html) and Primer3^34^ programs were used with default settings. The possible secondary structures such as homo-dimer, hetero-dimer, and hairpin were checked by using OligoAnalyzer™ Tool of Integrated DNA Technologies (IDT) (https://eu.idtdna.com/pages/tools/oligoanalyzer). In the selection of SARS-CoV-2 primers, attention was paid to the selection of genome regions that differ from other SARS-CoV relatives (NC_004718.3, AY613947.1, AY502927.1, AY278491.2, AY502924.1, and AY559094.1) (Supplementary Figs. S5 and S6). Therefore, the primers are specific to SARS-CoV-2 virus only and are expected to be free from possible cross reactions with other SARS viruses. Each gene-specific probe was labeled with different fluorescent dye: fluorescein amidides (FAM) for the viral *RdRp* gene, hexachloro-fluorescein (HEX) for the viral *N* gene, and carboxyrodamine (ROX) for the human *RP* gene. The concentration, size, and sequence of each primer or probe are indicated in Table 1.

**Table 1:** The sequence and concentration of primer and probe sets used in PCR reactions.

Primer/probe	Sequence (5′–3′)	Working conc. (µM)	Final conc. (µM)^a^	Size (bp)	Reference
*RdRp—*F	CCTCACTTGTTCTTGCTCGC	20	1	205	This study
*RdRp—*R	GCCGTGACAGCTTGACAAAT	20	1		
*RdRp—*Probe	FAM-GTGAAATGGTCATGTGTGGC-BHQ1	5	0.2		
*N–*F	TGAAACTCAAGCCTTACCGC	20	1	160	This study
*N—*R	TATAGCCCATCTGCCTTGTG	20	1		
*N—*Probe	HEX-ATCCATGAGCAGTGCTGAC-BHQ	5	0.2		
*RP—*F	AGATTTGGACCTGCGAGCG	20	1	92	Universal
*RP—*R	GATAGCAACAACTGAATAGCCAAGGT	20	1		
*RP—*Probe	ROX-TTCTGACCTGAAGGCTCTGCGCG-BHQ2	5	0.2		

^a^Final concentration represents the concentration of each oligonucleotide in reaction mixture (20 µL).

### Sample collection and RNA isolation

The RNA samples were extracted from nasopharyngeal swab or combined nasopharyngeal/oral swab collected from the patients at King Fahad Specialist Hospital (KFSH), Dammam, Saudi Arabia, between 01 and 30 November 2020. The swabs were collected in Virus Liquid Transport Medium-VTM per manufacture instruction for collecting and handling (Copan, U.S.A). Specimens were transported to the lab in cool box and kept refrigerated for not more than 8 h till time of nucleic acid extraction.

Prior to RNA extraction, collection tubes were vortexed; 200 or 400 µL of VTM were transferred to 2 mL tubes for RNA extraction using Magna Pure Compact (Roche, Germany) or ELITe InGenius (ELITechGroup, France) systems with elution set to 100 µL. Finally, 5 µL of extracted RNA was used as template for the rRT-PCR.

### rRT-PCR conditions

The following reaction mixture was prepared in a micro-centrifuge tube: 2 µL of 10× Buffer (Procomcure Biotech, Austria), 0.25 µL of dNTPs (10 mM each; Procomcure Biotech, Austria), 0.2 µL of uracil-DNA glycosylase (UDG) (1 U/µL; New England BioLabs Inc., USA), 0.25 µL of molecular grade dimethyl sulfoxide (DMSO, Sigma-Aldrich), primer and probe mixture (Molequle-On, New Zealand), 0.4 µL of VitaTaq HS polymerase (2 U/µL; (Procomcure Biotech, Austria), 0.5 µL of M-MuLV Reverse Transcriptase (200 U/µL) (New England BioLabs Inc., USA), 5 µL of RNA template and RNase/DNase-free ddH_2_O up to 20 µL.

The reaction mixture was transferred to a 96-well plate (MicroAmp™ Fast Optical 96-well Reaction Plate 0.1 mL, Applied Biosystems) and sealed with transparent optical film (MicroAmp™ Optical Adhesive Film, Applied Biosystems). *Pseudoviral* RNA containing the viral *RdRP* and *N* genes and human *RNaseP* (*RP*) mRNA sequences were used as positive control material (EURM-019, single stranded SARS-CoV-2 RNA fragments https://crm.jrc.ec.europa.eu/p/EURM-019). In negative control samples, RNase/DNase-free ddH_2_O was added instead of the RNA template.

Applied Biosystems™, 7500 Fast Real-Time PCR system was used for rRT-PCR reactions. Prior to this procedure, the instrument was calibrated using the Applied Biosystems™ 7500 Rapid Real Time PCR Systems Spectral Calibration Kit. The following conditions were applied for the rRT-PCR reaction: (1) reverse transcription at 42 °C for 15 min, (2) pre-denaturation at 95 °C for 5 min. For the cyclic reactions (40×) (3) denaturation at 95 °C for 5 s and (4) amplification at 60 °C for 30 s. The fluorescence reading was performed at the amplification step. The reporter dye channel was determined as FAM for the *RdRp* gene, VIC for the *N* gene, and ROX for the *RNAseP* (*RP*) gene. For the Applied Biosystems™ real time PCR instrument (7500 and StepOne models), "passive reference" is set to "none".

### Amplification efficiency and analytical sensitivity

The rRT-PCR amplification efficiency (E) was calculated for each viral gene. For this, dilution series of template RNA (Reference No: EURM-019, European Commission Joint Research Centre) was prepared and a standard curve was generated. Ct values were drawn for the logarithmic measurement of the template used. Amplification efficiency was calculated according to the following formula:

1 $${\text{E }} = { 1}00 \, \times \, \left( {{1}0^{{ - {1}/{\text{slope}}}} } \right).$$

The RNA concentration was determined by Nanodrop 2000 (Thermo Fisher Scientific, USA) and the copy number was determined according to the following formula^35,36^:

2 $${\text{RNA}}\,{\text{copies}}/{\text{mL }} = \, \left[ {{\text{RNA}}\,{\text{concentration }}\left( {{\text{g}}/{\text{mL}}} \right)/\left( {{\text{nt }}\,{\text{transcript}}\,{\text{length }} \times { 34}0} \right)} \right] \, \times { 6}.0{22 } \times { 1}0^{{{23}}} .$$

Multiple units are used to display limit-of-detection (LOD), for example viral genomic RNA copies per milliliter of transfer medium (copies/mL), copies/µL, copies per reaction volume, or molarity of the test target, etc., which are sometimes confused^37^. To demonstrate the sensitivity of current assay, the LOD number was calculated and expressed in two different units: (1) the number of copies in one µL of the reaction mixture (copies/µL) and (2) the number of copies in the reaction (copies/reaction). For this, serial dilution of synthetic RNA (5 × 10^4^, 5 × 10^3^, 5 × 10^2^, 5 × 10^1^, and 5 × 10^0^ copies/µL) was prepared. Since 5 µL of template solution was used in the PCR reaction, the lowest dilution (5 × 10^0^ copies/µL) contains 25 copies of RNA. In total, 25 copies of RNA in 20 µL of reaction mixture corresponds to 1.25 copies/µL or 25 copies/reaction. In addition, the probit regression analysis was performed by using MedCalc program (version 19.2; MedCalc Software, Ostend, Belgium).

### Diagnostic performance

To validate the new assay (called as mCoV-2), we performed rRT-PCR by using commercially available SARS-CoV-2 detection kits. For this purpose, the same RNA samples (n = 28) that were previously extracted from the COVID19 patients were used as the template. The reactions were run using either GeneFinder^TM^COVID-19 Plus RealAmp Kit (GeneFinder, Korea) or RealStar SARS-CoV-2 RT-PCR Kit 1.0 (Altona, Germany).

### Data analysis

Amplification curves of viral and human genes were considered to evaluate the results. After automatic adjustment of the cycle threshold (Ct or Cq) line, the representative Ct value of each gene was determined by using ABI 7500 software (v2.3). The positive cut-off value was set at cycle threshold number ≤ 37 with a sigmoidal curve. Any patient meets the criteria were accepted as positive.

### Ethical approval

The study is approved by the Institutional Review Board (IRB) at Imam Abdulrahman bin Faisal University (IAU) with an IRB number of IRB-2020-13-406. All methods were carried out in accordance with relevant guidelines and regulations. The de-identified samples left over after completion of diagnostic tests were used; hence this study requires no consenting as per institutional ethics committee regulations and informed consent.

## Results

### Standardization of the multiplex rRT-PCR

**Figure 1:**
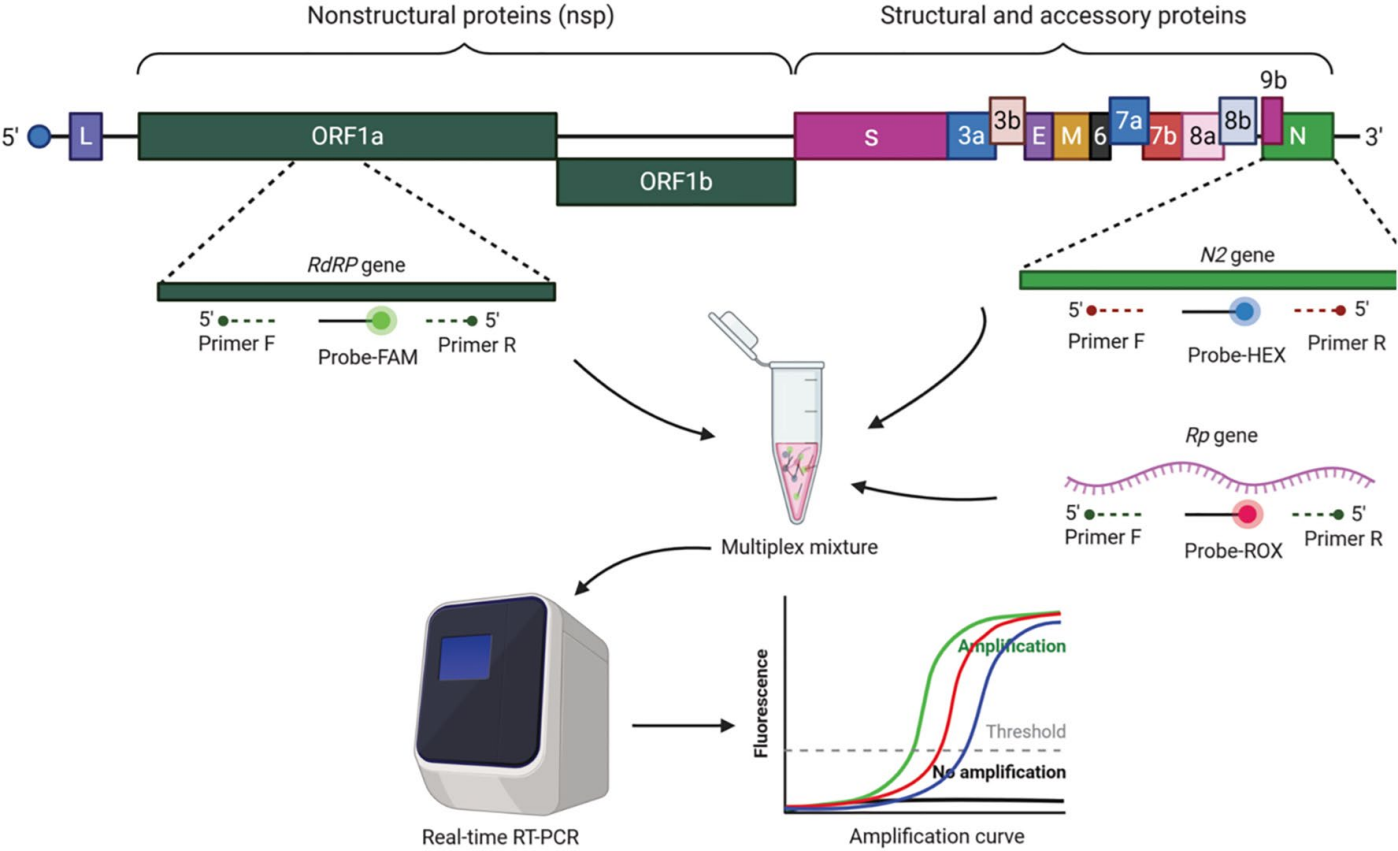
Genome structure of SARS-CoV-2 and the targeted genes in multiplex rRT-PCR assay.

A multiplex rRT-PCR assay was optimized for the diagnosis of SARS-CoV-2. The assay simultaneously targets two viral genes (*RdRP* and *N*) and one human gene (*RP*) as internal control (Fig. 1). The assay tested in 28 RNA samples collected from COVID-19 positive individuals. Supplementary Figure S1 exhibits the rRT-PCR outputs belonging to COVID-19 positive and negative individuals. In COVID-19 positive samples, the *RP*, *RdRP*, and *N* genes amplified simultaneously, forming S-shaped sigmoidal curves (Supplementary Fig. S1a). In the COVID-19 negative specimen, the internal control gene (*RP*) was the only gene amplified with a sigmoidal amplification curve (Supplementary Fig. S1b). In the positive control reactions, *pseudoviral* RNA including *N* and *RdRP* regions and a human *RP* mRNA was used as template. The amplification curves were obtained for all targeted genes (Supplementary Fig. S1c). In the negative control reactions, ddH_2_O was used as the template, which led no amplification line without primer dimer formation (homo-dimer or hetero-dimer). The results showed that the multiplex primer and probe design successfully amplify all targeted genes both in SARS-CoV-2 positive specimen and synthetic positive control samples without forming primer dimer or self-amplification.

**Figure 2:**
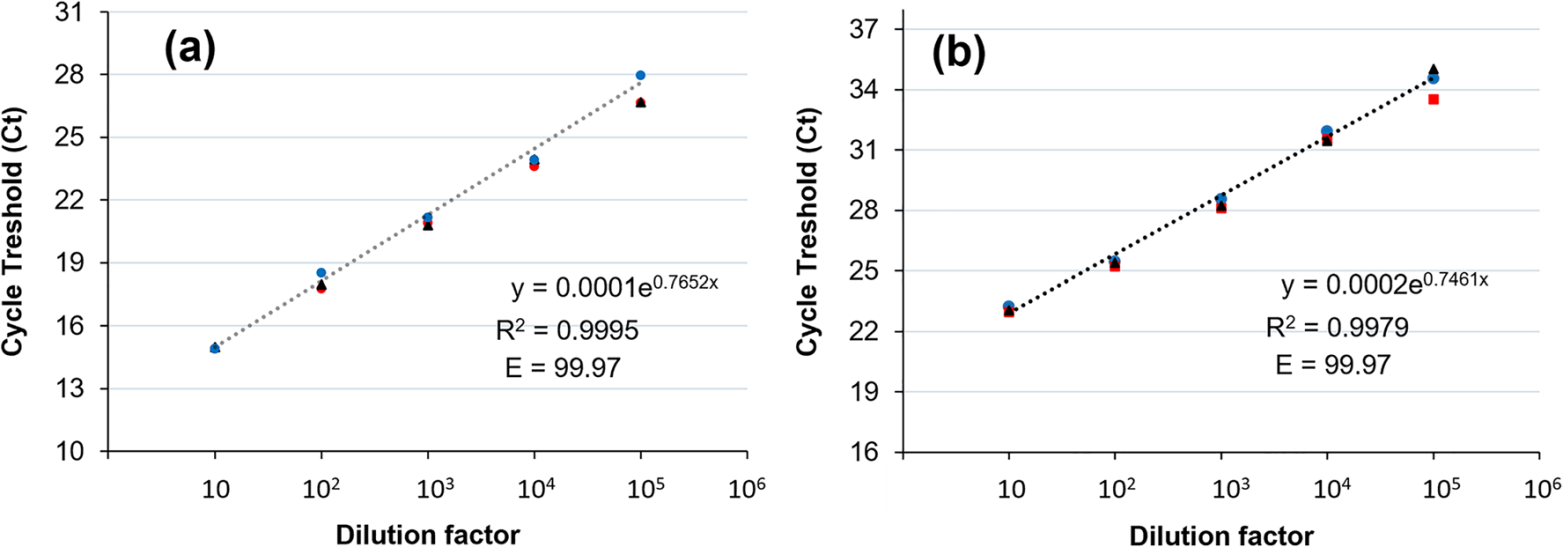
Standard curve analysis for multiplex rRT-PCR of (**a**) *RdRP* and (**b**) *N* primers. The template RNA was serially diluted with a range of 10^5^ to 10^1^. The reactions were carried out in triplicate. The amplification efficiencies (E) were shown on each graph. The error bars represent the standard deviation between the replicates. The error bars represent the standard deviation between the replicates. The amplification plots are shown in Supplementary Fig. S2.

In addition, the standard curve analysis was performed to test the sensitivity of the assay. For this purpose, a dilution series of clinical RNA was prepared with a dilution factor range of 10^5^ to 10^1^ (Fig. 2; Supplementary Fig. S2). Triplicate rRT-PCR analysis revealed that the results are consistent across technical replicates. The test ran successfully even on samples diluted 10^5^ times. The rRT-PCR efficiency for both *RdRP* and *N* genes is 99.7 and the R^2^ value is > 0.997, which shows the consistency and reliability of the assay.

### Validation of the assay

**Figure 3:**
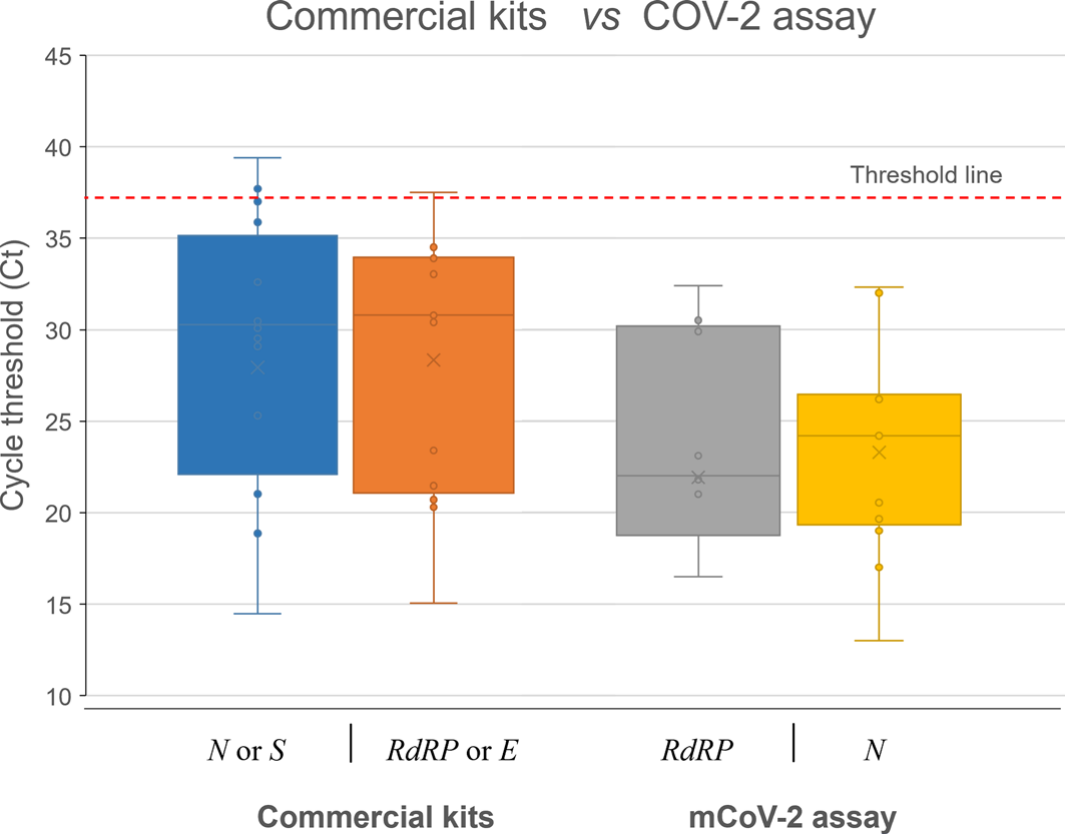
The cycle threshold (Ct) scores of the same clinical samples tested either the current mCoV-2 assay or commercial kits. Each bar represents different genes, which are *RdRP* (gray) and *N* (yellow) for mCoV-2 assay; and *N* or *S* (blue) and *RdRP* or *E* (orange) for commercial kits. Dashed line shows the positivity cut-off level equals to Ct 37.

The validation of the results has been performed by using two different commercially available kits (GeneFinder^TM^COVID-19 Plus RealAmp Kit (GeneFinder, Korea) and RealStar SARS-CoV-2 RT-PCR Kit 1.0 (Altona, Germany)) that are targeting different genes such as *RdRP*, *N*, *S*, and *E*. Among 28 clinically ‘confirmed’ SARS-CoV-2 positive samples, the current assay found 25 positives and three negatives (Supplementary Table S1). Accordingly, the Ct value equals and lower than 37 is accepted as positive. Besides, in both assays, the Ct score of those negative samples was higher than 37, which is out of the CDC and WHO recommendations^38,39^. Therefore, the samples having a Ct score of ≥ 37.01 are accepted as SARS-CoV-2 negative. In this case, the assay exhibited 100% positive percent agreement with those commercial assays. The distribution of Ct value obtained from both commercial methods and this mCoV-2 assay are displayed in Fig. 3. Since these kits target different genes, the Ct scores of those genes were combined. Accordingly, it is obvious that the average Ct value of the current assay is lower than those of the genes targeted in the comparative commercial kits. This result demonstrates the high sensitivity of the current assay.

### Limit-of-detection (LOD) and rRT-PCR efficiency

**Figure 4:**
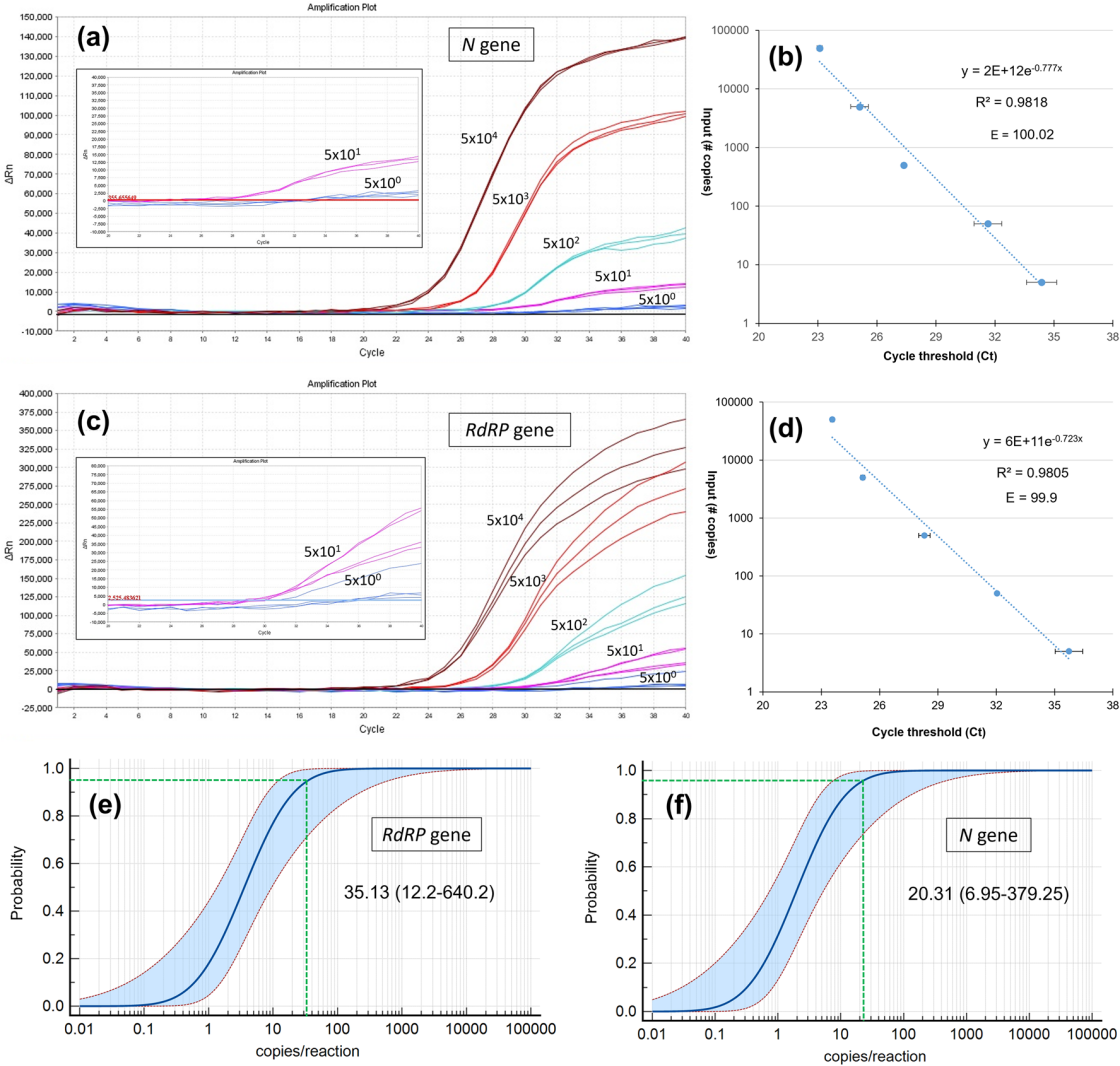
Determination of the limit of detection (LOD) for *RdRP* and *N* primers. The 5 × 10^4^ copy/µl *pseudoviral* RNA was serially diluted. The amplification plots (**a**,**c**) and the amplification efficiencies (E) (**b**,**d**) for *N* and *RdRP* genes were represented, respectively. The R^2^ value of the trendline and the efficiency (E) of the standard curve were displayed on each graph. The error bars represent the standard deviation between the replicates. The probit regression analysis of the LODs based on 10 technical replicates of serially diluted synthetic RNA: (**e**) *RdRP* and (**f**) *N* genes. Green dotted lines indicate LOD and values are denoted with 95% confidence intervals (CI) in parenthesis.

A serial dilution of synthetic RNA (5 × 10^4^, 5 × 10^3^, 5 × 10^2^, 5 × 10^1^, and 5 × 10^0^ copies/µL) was prepared to find out the limit-of-detection (LOD) for *RdRP* and *N* genes. The amplification plots, the amplification efficiencies (E), and R^2^ score are represented in Fig. 4. The probit regression analysis of the serially diluted synthetic RNAs (at least 10 replicates) determined the LOD of *N* gene as 0.81 copy/µL or 20.31 copy/reaction (Fig. 4e). Together with, the LOD of *RdRP* gene was computed as 1.40 copy/µL or 35.13 copy/reaction (Fig. 4f). The estimated 95% confidence intervals (CI) for the *N* and *RdRP* genes are between 6.95 and 379.25 and 12.2 and 640.2 copies/reaction, respectively. The standard curve analysis revealed that the E value of *N* and *RdRP* genes are 100.2 and 99.9, respectively. The R^2^ values are 0.9818 for the *N* and 0.9805 for the *RdRP* gene.

## Discussion

Since rRT-PCR method is considered the gold standard in the diagnosis of SARS-CoV-2, WHO and CDC recommend it as the diagnostic test for asymptomatic and mildly symptomatic patients^6,40^. However, rRT-PCR methods also have some drawbacks such as possible false-negative or false-positive results, the cost, etc^41^. In order to eliminate or minimize those drawbacks, multiplex rRT-PCR methods have been developed that target more than one gene at the same time. By doing this, it is aimed to improve rRT-PCR efficiency and sensitivity. Until now, many studies have been conducted to find the method that can detect the SARS-CoV-2 RNA with the highest sensitivity. For this, different combinations of targeted viral genes were tested in multiplex. According to the WHO recommendations, four viral genes (*RdRp*, *E*, *N* and *S* genes) can be used in multiplex rRT-PCR reactions in different combinations^42^. Along with these genes, primer and probe sequences of the human internal/positive control *RNase P* (*RP*) gene have been published and their use has been recommended by US CDC^24^. The studies to improve these protocols are still ongoing. Designing primers with the highest sensitivity towards the target gene, eliminating their cross reactivity, minimizing possible false negative and positive results, optimizing rRT-PCR conditions are examples of what can be improved by those studies. It is worth mention here that recently, Dekker et al.^43^ demonstrated a faulty design in *RP* primer sets defined by the CDC, which is another example of the importance of those improvement studies.

Together with, it has been shown by many studies that the SARS-CoV-2 genome evolve very fast as a consequence of the lack of proofreading activity of polymerases^29,44^. In a recent study, the nucleotide mutation rate of the SARS-CoV-2 genome was found as 6.677 × 10^−4^ substitution per site per year^28^. Due to the high susceptibility to mutation, it has been shown that more than one variant can be present in a patient at the same time^45^. For instance, a meta-transcriptome analyses of 110 SARS-CoV-2 sequences obtained from BAF samples (Bronchoalveolar fluid) from eight patients revealed the evolution of SARS-CoV-2 in the patient and that many variants can be found simultaneously in the individual^45^. Emergence of variations by new mutations, in particular when they placed on the RT-PCR primer binding sites, and active viral recombination^46^ can cause loss of the assay sensitivity^29^ and false-negative results^27^. Therefore, the selection and update of primer target regions and targeting more than one gene are of great importance. The RT-PCR protocols in particular the target regions/sequences should be updated by considering the most recent mutations and variants.

In this study, a multiplex rRT-PCR method has been developed that simultaneously targets the viral *N* and *RdRP* genes and the human *RP* gene. The primer and probe sets were designed to obtain the best PCR efficiency and target specificity. Due to the rapid evolution of SARS-CoV-2 genome^47^, attention was paid to designing the primers that are covering the most recent variant of concerns (VOC) such as alpha (B.1.1.7), B.1.351 (Beta), P.1 (Gamma), and B.1.617.2 (Delta). For this purpose, 344 SARS-CoV-2 genomes from different lineages and locations (including all continents) were screened and the primer binding sites were defined as the most conserved regions (Supplementary Figs. S3 and S4). In addition, to improve the specificity, the oligos were set to the variable region between SARS-CoV-2 and other SARS viruses (Supplementary Figs. S5 and S6). A recent genome analysis of 31,000 SARS-CoV-2 sequences from nasopharyngeal samples of 30 patients showed that 99% of the genome regions targeted by the RT-PCR primers were identical^48^. On the other hand, 1% of heterogeneous sequences presented inconsistencies, particularly a mismatch between the SARS-CoV-2 genome and the commercial primer, including genes selected in WHO-recommended RT-PCR detection tests. For example, two regions of genetic variability were identified in the sequence of *RdRP* gene primers recommended by the US CDC^48,49^. This variation can have a critical impact on the reliability and sensitivity of the assay.

According to the results, the rRT-PCR efficiencies of tenfold dilutions series of the standards were > 99 for both *N* and *RdRP* genes (Fig. 2; Supplementary Fig. S2), which matches the criteria for an efficient RT-qPCR assay^50^. Besides, the current protocol allows the diagnosis of SARS-CoV-2 RNA with a limit of detection (LOD) value of 0.81 copies/µL or 20.31 copies/reaction for the *N* gene, and 1.40 copies/µL or 35.13 copies/reaction for the *RdRP* gene (Fig. 4). The estimated 95% confidence intervals (CI) for the LODs of *N* and *RdRP* genes are 6.95–379.25 and 12.2–640.2 copies/reaction, respectively. The corresponding copy number of each gene per µL (copies/µL) varied between 0.27 and 15.17 copies/µL for the *N* gene, and 0.48 and 25.60 for the *RdRP* gene. The LOD of the CDC’s 2019-nCoV Real-Time RT-PCR Diagnostic Panel is 10 copies/μL. According to Vogels et al.^51^, all SARS-CoV-2 primer and probe sets that has capacity to detect 500 copies/reaction can be used to diagnose SARS-CoV-2. Pfefferle et al.^52^ demonstrated that the LOD of RT-PCR assay was 689.3 copies/mL with 275.72 copies per reaction. In this study, GeneFinder^TM^COVID-19 Plus RealAmp Kit (GeneFinder, Korea) and RealStar SARS-CoV-2 RT-PCR Kit 1.0 (Altona, Germany) has been used to verify the assay control. The LOD of the GeneFinder kit is 0.5 copies/µL for both upper respiratory and sputum specimen as measured on the ABI 7500, ABI 7500 Fast, and CFX96 instruments^53^. Another study using a digital PCR (dPCR) assay found it as 0.149 and 0.163 copies/µL for *N1* and *N2* genes, respectively^54^. The LOD of the RealStar kit was 1.2 copies/µL in nasopharyngeal swabs (NP) specimens; but 12 copies/µL in bronchoalveolar lavage (BAL) specimens^55^. The LODs of three SARS-CoV-2 RT-PCR kits, Allplex (Seegene, Korea), PowerChek (KogeneBiotech, Korea), and Real-Q (BioSewoom, Korea), were found to be 153.94, 84.12, and 80.60 copies/reaction, respectively^56^. Overall, it can be said that the current assay has at least as high sensitivity as the recommended and known tests, or even more than some.

The validation of the assay was tested by using viral RNA samples extracted from the swabs of SARS-CoV-2 positive individuals (n = 28). It is revealed that three samples out of 28 did not match with the results of the commercial kits (Supplementary Table S1). In both assays, either commercial or the current one, it is estimated that the Ct score of those negative samples was higher than > 37.01 which is out of the WHO recommendation^36^. Therefore, in the current mCoV-2 assay, they are called as negative. Accordingly, the Ct value equals and lower than 37 is accepted as positive.

For COVID19, RT-PCR detection kits are commonly destined to amplify the genes *S, E, N, RdRP, and ORF1a/b,* but *ORF1a/b* and *E* were mostly applied^57,58^. In China, Orf*1ab* and *N* genes are regularly used, while *N1, N2 and N3* genes were utilized in US CDC and *E, N,* and *RdRP* genes in Europe^59^. The importance of *N1* and *N2* primer-probes is for providing a less conservative but more sensitive than the *RdRP* primer-probes especially in samples that have low viral titers^60,61^. In a recent study, where swabs from confirmed cases were taken from nasopharynx and pharynx targeting *ORF1ab* and *N* genes yielded the best sensitivity when compared to positive confirmed samples^61^. Chu et al.^62^ have reported two assays that had the capability to achieve a large dynamic range and recommended targeting *N* gene for screening and the *ORF1b* gene to confirm the result. Together with, a case report found that after 9 and 10 days from the onset of disease, an RT-PCR kit detected a single positive gene which is *nucleocapsid* (*N*), but not the orf1ab gene. On day 16, both genes resulted in positive amplification which suggests that the *N* gene is more sensitive to amplify than the *orf1ab* gene^63^. The abovementioned studies confirmed the capability of targeting *N* gene to be utilized in the detection of COVID-19 with other genes. While Li et al.^64^ concluded that the more stable *E* gene is the target for the standardization of coronavirus tests, but *N and RdRP* genes are mostly targeted to confirm the results. In our study, it seems that targeting *RdRP* and *N* genes will make the test more sensitive. However, more studies are needed for fast and accurate COVID19 detection.

## Conclusion

Due to the spread of COVID-19 all over the world and emergence of new SARS-CoV-2 variants, there is an urgent need to develop more reliable and sensitive methods and to improve existing methods. Establishing sensitive primers and PCR conditions are extremely important to detect SARS-CoV-2 early and to control the spread of the disease. Current study describes a multiplex rRT-PCR assay that simultaneously targets two viral (*RdRP* and *N*) and one human internal control gene (*RP*). In addition, the experimental design is free from background (self- or hetero-dimer formations) with high sensitivity. Thanks to this strategy, fast, reliable, and easy-to-use rRT-PCR method is obtained to detect SARS-CoV-2. The sensitivity of the method should be evaluated in different RT-PCR devices.

## Supplementary Information

Supplementary Figure S1.

Supplementary Figure S2.

Supplementary Figure S3.

Supplementary Figure S4.

Supplementary Figure S5.

Supplementary Figure S6.

Supplementary Legends.

Supplementary Table S1.
