# Supplementary figures and images for: Multiplex real-time RT-PCR method for the diagnosis of SARS-CoV-2 by targeting viral N, RdRP and human RP genes
Source: Sci Rep. 2022 Feb 18;12:2853. doi: 10.1038/s41598-022-06977-z (PMC8857243; doi:10.1038/s41598-022-06977-z)

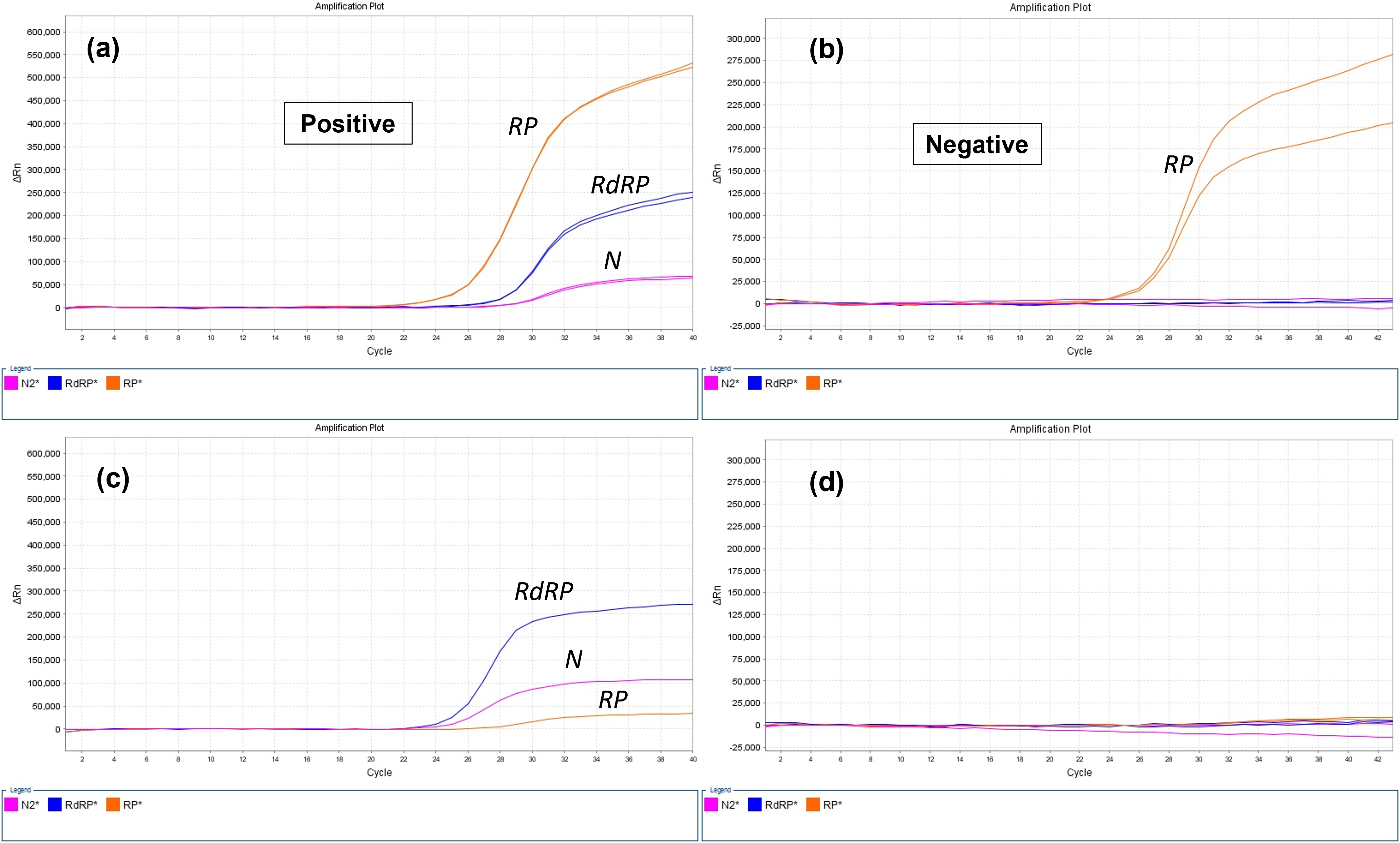

Supplement: Supplementary file 1 — Supplementary Figure S1. [file 41598_2022_6977_MOESM1_ESM.png]

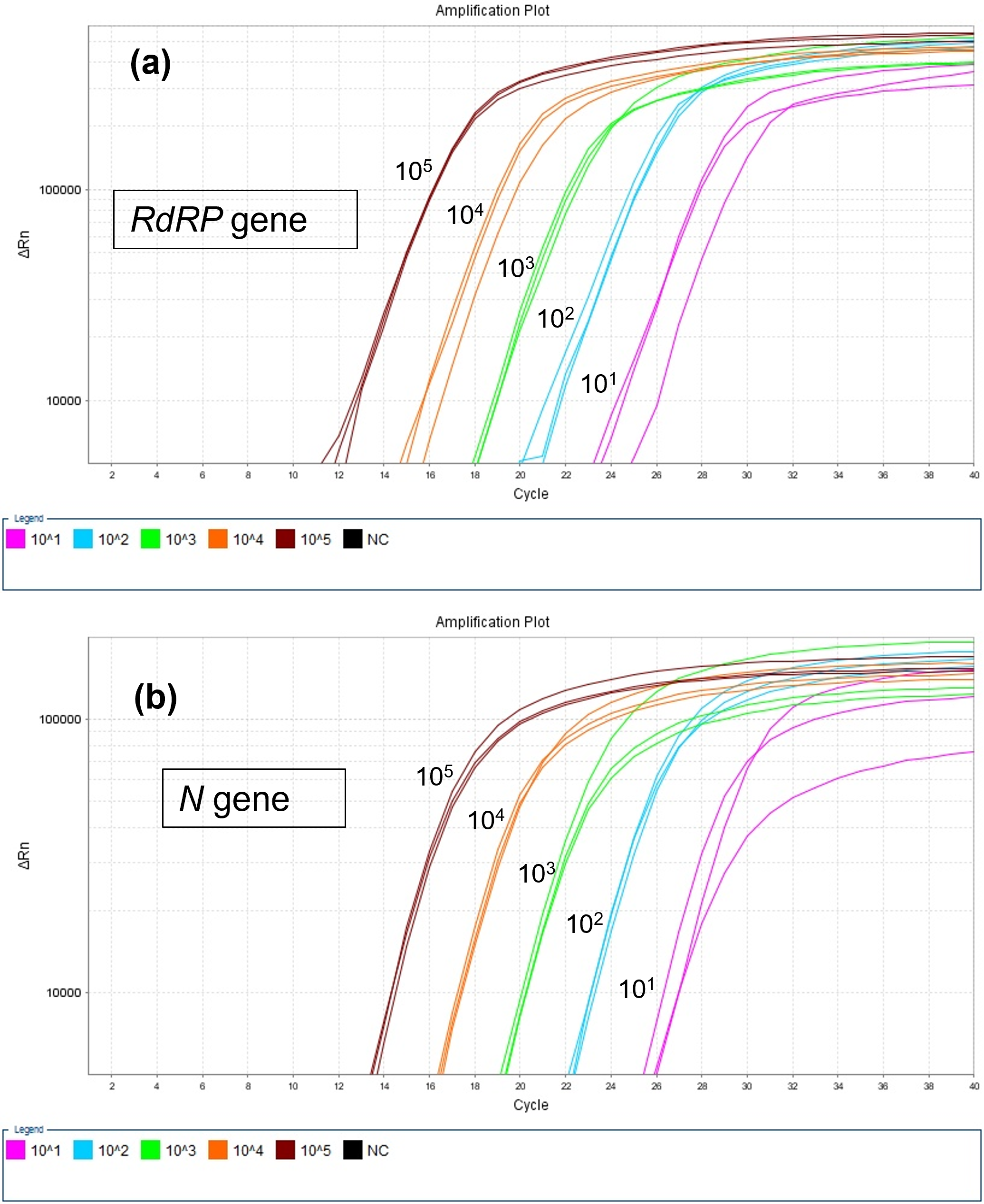

Supplement: Supplementary file 2 — Supplementary Figure S2. [file 41598_2022_6977_MOESM2_ESM.png]

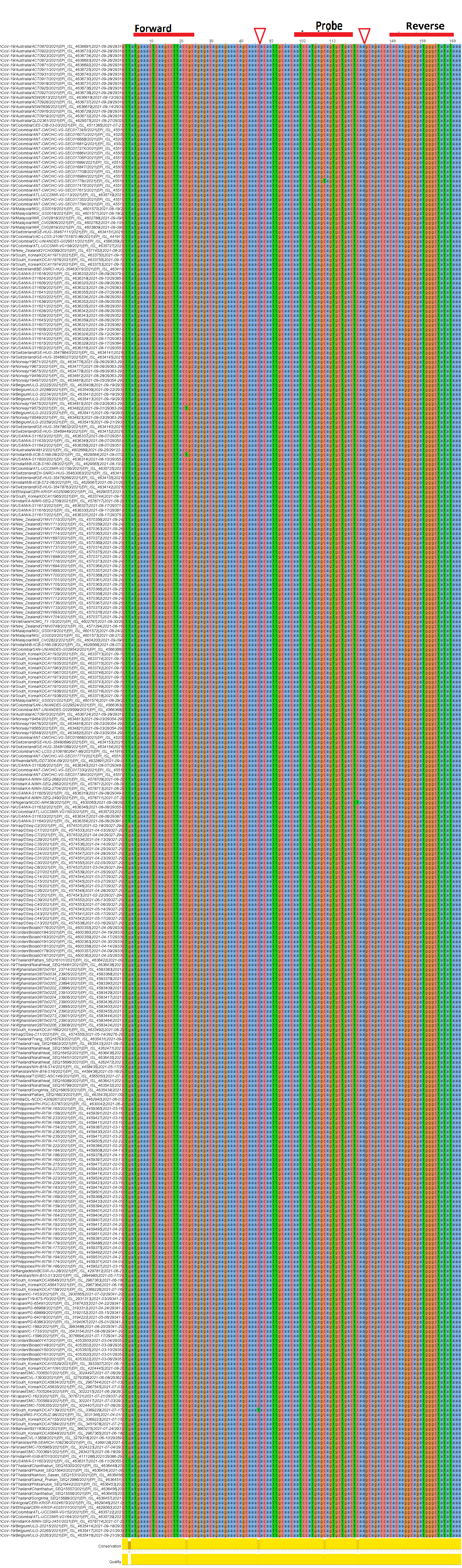

Supplement: Supplementary file 3 — Supplementary Figure S3. [file 41598_2022_6977_MOESM3_ESM.png]

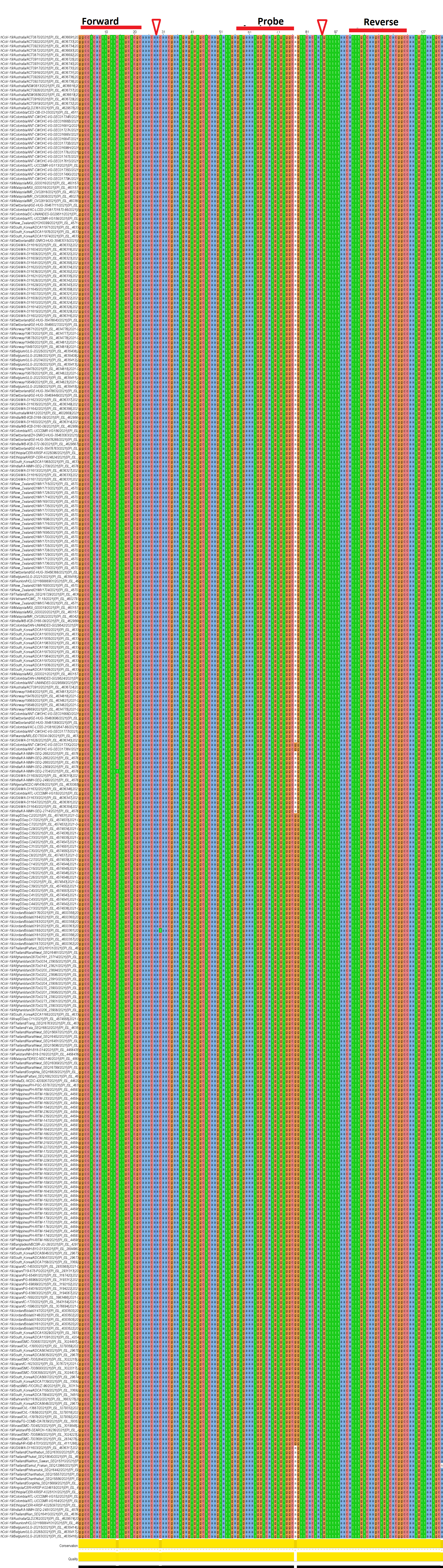

Supplement: Supplementary file 4 — Supplementary Figure S4. [file 41598_2022_6977_MOESM4_ESM.png]

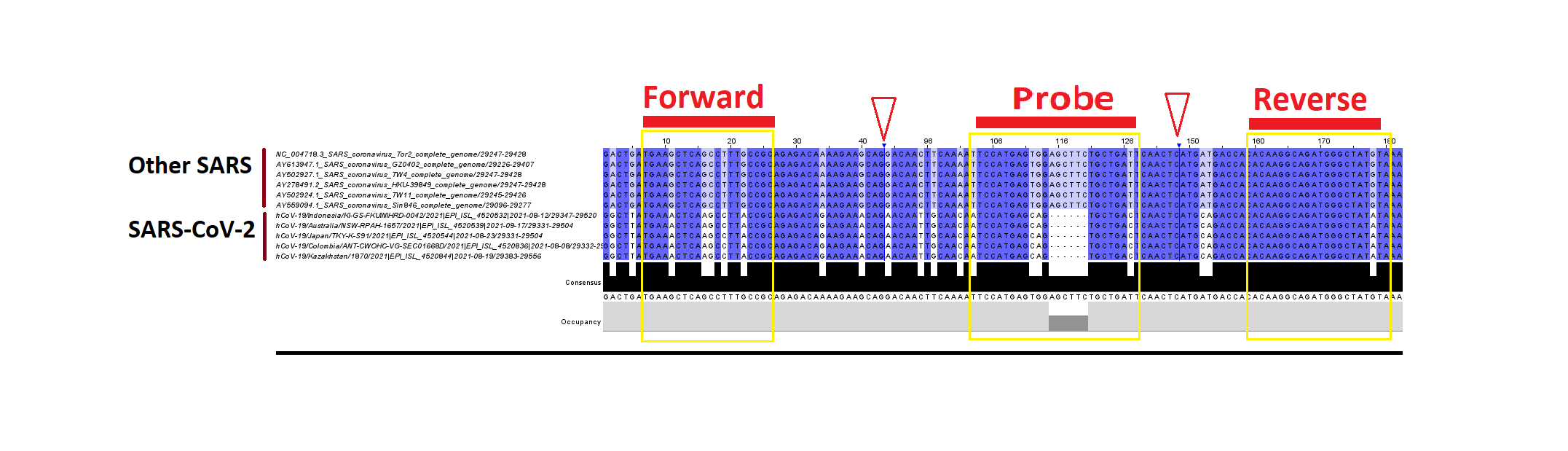

Supplement: Supplementary file 5 — Supplementary Figure S5. [file 41598_2022_6977_MOESM5_ESM.png]

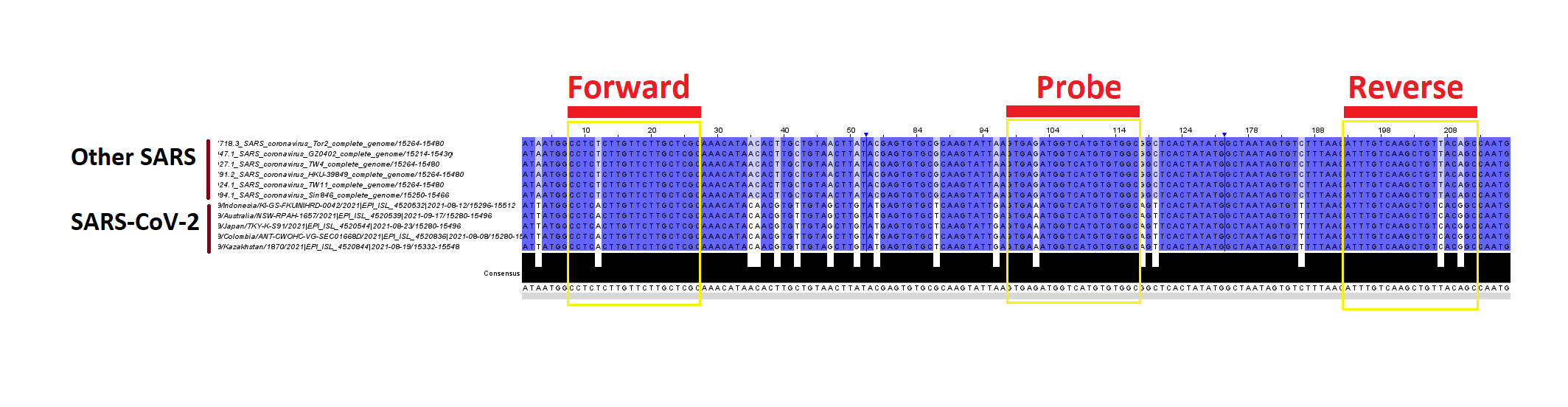

Supplement: Supplementary file 6 — Supplementary Figure S6. [file 41598_2022_6977_MOESM6_ESM.png]
