# Supplementary material for: Multiplex real-time RT-PCR method for the diagnosis of SARS-CoV-2 by targeting viral N, RdRP and human RP genes
Source: Sci Rep. 2022 Feb 18;12:2853. doi: 10.1038/s41598-022-06977-z (PMC8857243; doi:10.1038/s41598-022-06977-z)
Supplement: Supplementary file 7 — Supplementary Legends. [file 41598_2022_6977_MOESM7_ESM.docx]

**Supplementary Figure captions**

**Figure S1.** Amplification curves of clinical samples belongs to **(a)** SARS-CoV-2 `positive` and **(b)** SARS-CoV-2 `negative` specimens. The amplification curve of **(c)** positive control and **(d)** negative control.

**Figure S2.** The amplification plots of *RdRP* and *N* genes. A dilution series of clinical RNA was prepared with a dilution factor range of 10^5^ to 10^1^. NC, negative control.

**Figure S3.** Alignment of 344 *RdRP* sequences belongs to SARS-CoV-2 including alpha (B.1.1.7), B.1.351 (Beta), P.1 (Gamma), and B.1.617.2 (Delta) variants collected all around the world. The primer and probe binding sites exhibited 100% conservation. The arrowhead shows the hided regions.

**Figure S4.** Alignment of 344 *N* sequences belongs to SARS-CoV-2 including alpha (B.1.1.7), B.1.351 (Beta), P.1 (Gamma), and B.1.617.2 (Delta) variants collected all around the world. The primer and probe regions exhibited 100% conservation. The arrowhead shows the hided regions.

**Figure S5.** The primer and probe binding sites of *N* gene for the SARS-CoV-2 and other SARS viruses. The arrowhead shows the hided regions. The reference IDs for the other SARS are NC_004718.3, AY613947.1, AY502927.1, AY278491.2, AY502924.1, and AY559094.1.

**Figure S6.** The primer and probe binding sites of *RdRP* gene for the SARS-CoV-2 and other SARS viruses. The reference IDs for the other SARS are NC_004718.3, AY613947.1, AY502927.1, AY278491.2, AY502924.1, and AY559094.1.
