# Supplementary material for: Multiplex real-time RT-PCR method for the diagnosis of SARS-CoV-2 by targeting viral N, RdRP and human RP genes
Source: Sci Rep. 2022 Feb 18;12:2853. doi: 10.1038/s41598-022-06977-z (PMC8857243; doi:10.1038/s41598-022-06977-z)
Supplement: Supplementary file 8 — Supplementary Table S1. [file 41598_2022_6977_MOESM8_ESM.pdf]

Table S1. The Ct values and results from the commercial and current assays in clinical samples.

	Commercial					mCOV-2 assay			
Gene / Sample	*RdRP*	*N2*	*E*	*S*	Result*	*N2*	*RdRP*	*RP*	Result*
S1	29.1	30.79	37.75		positive	29.9	26.5	23.2	positive
S2	39.4	ND	41.02		negative	ND	ND	25.2	negative
S3	29.53	33.04	30.41		positive	30.5	26.2	30.4	positive
S4	30.46	33.37	30.55		positive	34	26.4	24	positive
S5	32.9	ND	32.06		positive	ND	24.2	20.1	positive
S6			15.07	14.47	positive	16.5	13	23	positive
S7			34	32.78	positive	34.2	32	21	positive
S8			21.48	21.02	positive	21.8	19.8	24.2	positive
S9			33.9	35.88	positive	36.4	26.2	20.3	positive
S10			20.7	30.08	positive	22	17	19.8	positive
S11			20.3	18.87	positive	21	19	22.5	positive
S12	32.6	30.4	34.5		positive	30.1	19.64	25	positive
S13	37.2	37.5	ND		negative	ND	ND	26	negative
S14	25.3	23.4	25.6		positive	23.1	20.53	18.2	positive
S15	37.7	ND	37.9		negative	37.1	38.4	29.4	negative
S16	25	26.4	25.4		positive	22.4	24.1	26	positive
S17	35.2	36.4	32.4		positive	32.7	34.6	24.2	positive
S18	26.8	24	28		positive	27.4	26.5	22.5	positive
S19	35.4	31.2	30.1		positive	34.1	36.4	23	positive
S20	35.5	35.7	29.5		positive	35.6	34.9	27.5	positive
S21	33.8	32.9	35		positive	36	34.5	31.5	positive
S22	24.4	26.3	28.7		positive	16.1	20.2	18.8	positive
S23	35.7	ND	34		positive	33.1	35	34.2	positive
S24			33.4	36.6	positive	35.4	33	35	positive
S25			31.6	36.2	positive	35.8	36.4	31.2	positive
S26			23.5	31.2	positive	23.4	27	23	positive
S27			35.5	35.4	positive	31.4	35.8	26.3	positive
S28			29.9	27.1	positive	27.6	25.8	24.1	positive

ND: not determined.

* the samples having a Ct score higher than 37.01 was accepted as `negative`.
